# Peri-Operative Care

**DOI:** 10.3390/healthcare9070790

**Published:** 2021-06-23

**Authors:** John B. Kortbeek

**Affiliations:** Departments of Surgery, Anaesthesia and Critical Care Cumming School of Medicine, South Health Campus, University of Calgary, 4448 Front ST SE, Calgary, AB T3M1M4, Canada; kortbeek@ucalgary.ca

In the history of surgery, 1911 was a sentinel year. In that year, Ernest Codman resigned his staff position at the Massachusetts General Hospital to found the “End Result Hospital”. He was committed to improving the quality of care of his surgical patients through careful observation and measurement. Codman was a founding member of the American College of Surgeons [1]. He understood that even the very best can err. His goal of revolutionizing surgical care through a better appreciation of patient outcomes and applying this knowledge to improve the delivery of surgical care was prescient. Unfortunately for Codman, he was a century ahead of his time and his ideas were met with ridicule and derision. I suspect that Codman would be both gratified and impressed to see the transformation that has occurred over the past several decades and the excellent work being presented in this Special Issue on perioperative care.

Olle Ljungqvist, a Swedish surgeon, and a group of like-minded surgeons from Denmark, the UK, and the Netherlands, founded the enhanced recovery after surgery study group in 2001. They were convinced, based on published studies of fast-track surgery and enhanced recovery programs, that there was a tremendous opportunity to improve outcomes in surgery through standardization, measurement, and feedback. The enhanced recovery after surgery (ERAS) era was born [2]. Studies incorporating these systems have demonstrated improvements in the length of stay and reductions in morbidity of 20–50%.

In this issue, Pough and colleagues explore variations in antibiotic administration and compliance with prophylaxis standards and identify opportunities for further reductions in surgical site infection in pediatric colorectal surgery populations [3]. They identify an approach to address these consistent with ERAS^®^ protocols. Johnson and associates describe the methodology they will employ to harness the power of the ERAS databases from two large ERAS programs. They will expand the pharmacologic data retrieval to propose further enhancements that may reduce the important morbidities of SSI, thromboembolism, and postoperative nausea and vomiting [4]. Finally, Hasan et al. take the ERAS concept one step further in describing a Peri-operative Surgical Home in pediatrics [5]. The proposed concept was conceived during the current COVID-19 pandemic, with its devastating impact on scheduled surgeries and the need for greater coordination to mitigate the impact of COVID-19, and to streamline recovery as we emerge from the pandemic. The study outlines the potential benefits of enhanced perioperative multidisciplinary coordination.

Crisis resource management (CRM) grew from its origins in aviation, following major airline disasters, and the development of crew resource management. Anaesthesiologists were the early adopters in medicine, but CRM subsequently spread to surgery, trauma, critical care, emergency medicine, and other disciplines. The important and central role of both planning and simulation to successfully implement CRM strategies have come to be generally accepted and described [6]. Ebbitt et al. describe the development of a CRM for the rare but challenging critical event of malignant hyperthermia [7]. The essential elements of the planned response, team leadership, roles, and equipment are described in detail. An illustrative case report allows readers to live the experience.

What operation and when to perform it are questions as old as surgery. One of the fathers of surgery, Theodor Billroth, was both an exemplary teacher and a scholar, who sought to record his outcomes. His goal was to have a basis for recommending the correct surgery for his patients [8]. His legacy lives on in many ways, but perhaps none are more iconic than the classic Billroth 1 and Billroth 2 operations of upper gastro-intestinal surgery. This sentinel dilemma, what is the right procedure, has only grown with the burgeoning number of possible operations supported by modern medical science. In this issue, Bacusca et al. perform a meta-analysis on the choice between performing or not performing a prophylactic donor heart tricuspid annuloplasty in an orthotopic heart transplantation [9]. Their results are suggestive, but a definitive answer, once again, awaits a properly performed randomized clinical trial.

Charles Wilson wrote a wonderful opinion piece on the future of sensors in medicine in 1999 [10]. He predicted many of the forthcoming advances in closed loop devices, biosensors, and smart drugs. He noted that significant improvements in the quality of care will accompany these technologies and surmised that the organization and delivery of healthcare will evolve and change as a result. He had an uncanny ability to foresee coming events, illustrated by the article’s closing quote advocating for, “New and better vaccines for preventing common conditions afflicting many millions throughout the world…”. In this issue, Restrepo and colleagues present us with a 2021 update on the status of biosensor development, with early experimental application in a critical care unit [11].

Hyland and associates include a wonderful, thorough, and comprehensive review of the use and stewardship of opiates and perioperative pain management in the 21st century [12]. The article should be required reading for surgical trainees as they navigate the changing landscape of managing pain in surgical patients. The paper summarizes approaches that employ all of the available strategies when navigating the complex waters posed by patients with opiate exposure or dependency. Patients with co morbidities, and the increasing number of patients regularly using cannabis or opioid agonists and antagonists are also addressed. Ensuring the best peri-operative experience while mitigating the risks of chronic opioid dependence are quality expectations of modern surgery programs.

The ERAS and strategies to standardize and inform surgical care have become expected norms. This does not make them easy to adopt and implement. Fortunately, Lovely and Larson have provided a road map for success and describe the dos and do not learned from many of our colleagues along the way [13].

I hope that you enjoy this Special Issue of *Healthcare* focusing on peri-operative quality and safety. Ernest Codman would have been pleased.

## Data Availability

Not applicable.
